# The nature and prevalence of threats to medical student placement capacity in primary care: a survey of East of England GP practices

**DOI:** 10.3399/BJGPO.2022.0127

**Published:** 2022-11-30

**Authors:** Richard Darnton, Sam Amey, James Brimicombe

**Affiliations:** 1 Department of Public Health and Primary Care, University of Cambridge, Cambridge, UK; 2 School of Clinical Medicine, University of Cambridge, Cambridge, UK

**Keywords:** practice organisation, undergraduate education, students, medical, teaching, general practice, primary healthcare

## Abstract

**Background:**

GP practices deliver vital medical student teaching in the face of increasingly challenging circumstances.

**Aim:**

To understand the nature and scale of threats to medical student teaching capacity in primary care.

**Design & setting:**

An electronic survey of a predefined population of 120 East of England GP practices that host medical student placements.

**Method:**

The survey was completed on behalf of the practice by the GP lead for medical student teaching. They were asked to pick (from a list of 16) the four main challenges they faced delivering medical student teaching placements, then explain their selection and suggest solutions. Thematic analysis of free-text responses was undertaken from an activity theory perspective.

**Results:**

Responses were received from 114 of the 120 practices in the study population (95% response rate). The most commonly selected challenges to delivering placements were clinical/practice workload (picked by 92 practices), and lack of space in the practice (picked by 63 practices). Thematic analysis produced a model whereby a practice’s decision to continue hosting students was influenced by level of motivation and burden of teaching, but only if a certain level of resource enablement is present. Analysis of free-text responses suggested that space pressures were perceived as being exacerbated by the need to accommodate more clinicians, especially advanced practitioners employed by primary care networks (PCNs) under the additional roles reimbursement scheme (ARRS).

**Conclusion:**

This study provides much-needed quantitative evidence to support the view that lack of space in GP premises is a major threat to the future of undergraduate general practice.

## How this fits in

Medical student placements in general practice are vital for the future of the primary care workforce. It was found that after clinical/practice workload, lack of premises space currently appears to be the biggest threat to medical student teaching in GP practices. This seems to have been exacerbated by the increased need to accommodate other learners and more clinicians (particularly advanced practitioners employed by PCNs). Without urgent national action to address the severe shortage of space in GP premises, medical student teaching capacity may be lost, resulting in a 'death spiral' of ever-worsening GP recruitment and placement capacity.

## Introduction

In the UK, medical student teaching capacity in primary care is a matter of national concern.^1^ This is because GP practices provide vital teaching capacity for medical schools^2^ and because the degree of medical student exposure to authentic general practice is associated with their likelihood of choosing it as a career.^3^ Given the intense pressures currently facing UK GP practices, it is important to understand the nature and the scale of the challenges they face delivering medical student placements. While studies have identified a range of facilitators and barriers to general practice teaching,^4^ they do not provide evidence on the scale or prevalence of the different challenges to primary care placement capacity. Furthermore, these studies took place before the COVID-19 pandemic (that is, pre-2020), and since then the delivery of UK primary care (and the teaching provided in this environment^5^) has changed significantly. For example, there has been a sustained increase in the use of remote consulting,^6^ primary care is facing unprecedented workload pressures,^7^ PCNs (created in 2019) have matured,^8^ and GP trainee numbers have increased by 25% since 2019.^9^ There is therefore a need to understand the current threats to medical student teaching capacity in general practice both in terms of their prevalence and their nature. Hence, a mixed-methods survey of a defined population of GP teaching practices was undertaken in order to address this need.

## Method

### Survey instrument

An electronic survey instrument was administered to a defined population of GP practices using the Qualtrics survey platform. Practices were asked to pick, from a list of 16, the four main challenges they faced delivering medical student placements. This list had been developed through an iterative piloting and refinement process with an advisory group of GP educators. One of the 16 options was 'other' and this allowed for responders to specify using free text. The list of options was randomly ordered for each responder. Two free-text questions followed: one asking responders to explain the choice they made from the list; and one asking responders about possible solutions to the challenges they picked. The survey contained some other questions (regarding the shift to remote consulting) and these data are reported elsewhere.^10^ A copy of the survey is available in Supplementary Box S1.

### Study population

The study population was defined as all GP practices that had, at any time between 1 January and 31 March 2022, hosted Cambridge medical students studying years 4, 5, or 6 of the curriculum (*n* = 120). This is a highly heterogenous group of practices (in terms of size, staffing, location, and population served) and it is widely distributed across the East of England. The survey was completed by the doctor with lead responsibility for overseeing the practice’s undergraduate medical teaching activity.

### Data analysis

Quantitative data were analysed using simple descriptive statistics. Qualitative data (free-text responses) were analysed thematically from an activity theory perspective.^11^ The method of thematic analysis is described in Box 1 and followed the method outlined by Kiger and Varpio.^12^

Box 1 **Detailed description of the process of thematic analysis used**

1. RD read the dataset, noting key points and quotes as well as first impressions of meanings, themes, and interrelationships arising from across the dataset.
2. RD used these notes to develop a coding framework arising from the data and informed by the theoretical perspective.
3. SA tested this framework by coding a portion of the dataset looking for coding problems, ambiguities, and potential new codes.
4. RD and SA reviewed and revised the coding framework in the light of this testing. SA then coded the entire dataset.
5. RD examined the coded data for themes and interrelationships, organising them where possible into a thematic map.
6. RD and SA independently reviewed these themes with respect to the coding to check whether they were adequately supported by the data. In the light of this, themes and their interrelationships were retired or revised.
7. Themes were then grouped into major overarching themes and hierarchies, which were iteratively revised and refined until they represented the essence of the dataset.

## Results

A total of 114 out of the study population of 120 practices completed the picking list question, giving a 95% response rate. Of these practices, 71 also provided free-text answers to one or both of the open ended follow-on questions. Many of these answers were extensive, comprising three or more sentences.

### Quantitative data: frequency with which each factor was picked

**Figure 1.**
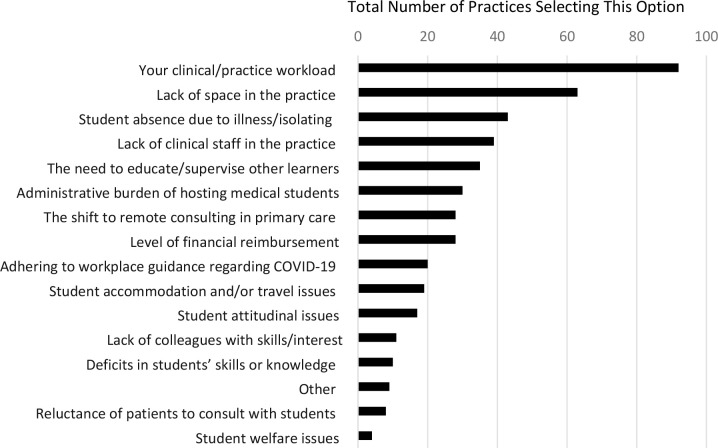
Main challenges of delivering medical student placements faced by practices (*n* = 114 [each practice selected four options])

From the list of challenges to hosting student placements, clinical/practice workload and lack of space in the practice were by far the most commonly selected options (*n* = 92/114 and *n* = 63/114, respectively). Ranked below, the next most frequently picked challenges were student absence owing to illness or isolating (*n* = 43/114 practices), lack of clinical staff in the practice (*n* = 39/114 practices), and the need to educate other learners hosted by the practice (*n* = 35/114 practices). The 'other' option was picked by only nine out of 114 practices, and did not consistently produce any answers that lay outside of the 15 predefined options. Figure 1 and Table 1 summarise the frequencies with which each of the options were picked.

**Table 1.** Frequencies with which each of the options were picked

Challenges faced delivering medical student placements (each of the 114 practices selected four options from this list)	Practices selecting this option, *n*
Your clinical/practice workload	92
Lack of space in the practice	63
Student absence owing to illness or isolating	43
Lack of clinical staff in the practice	39
The need to educate or supervise other learners that are hosted by the practice	35
Administrative burden of hosting medical students	30
The shift to remote consulting (that is, telephone and/or video) in primary care	28
Level of financial reimbursement for hosting medical students	28
Adhering to workplace guidance (for example, local and/or national policies) regarding COVID-19	20
Student accommodation and/or travel issues	19
Student attitudinal issues	17
Lack of colleagues with skills and/or interest in supervising medical students	11
Deficits in students’ skills or knowledge	10
Other	9
Reluctance of patients to consult with students	8
Student welfare issues	4

### Qualitative data: thematic analysis of free-text answers

Thematic analysis of the qualitive data from an activity theory perspective^11^ elicited the following three major themes:

1. factors that enabled or prevented practices to host students ('enablement');
2. factors that influenced a practice’s motivation to host students;
3. factors that influenced the burden of hosting students.

**Figure 2.**
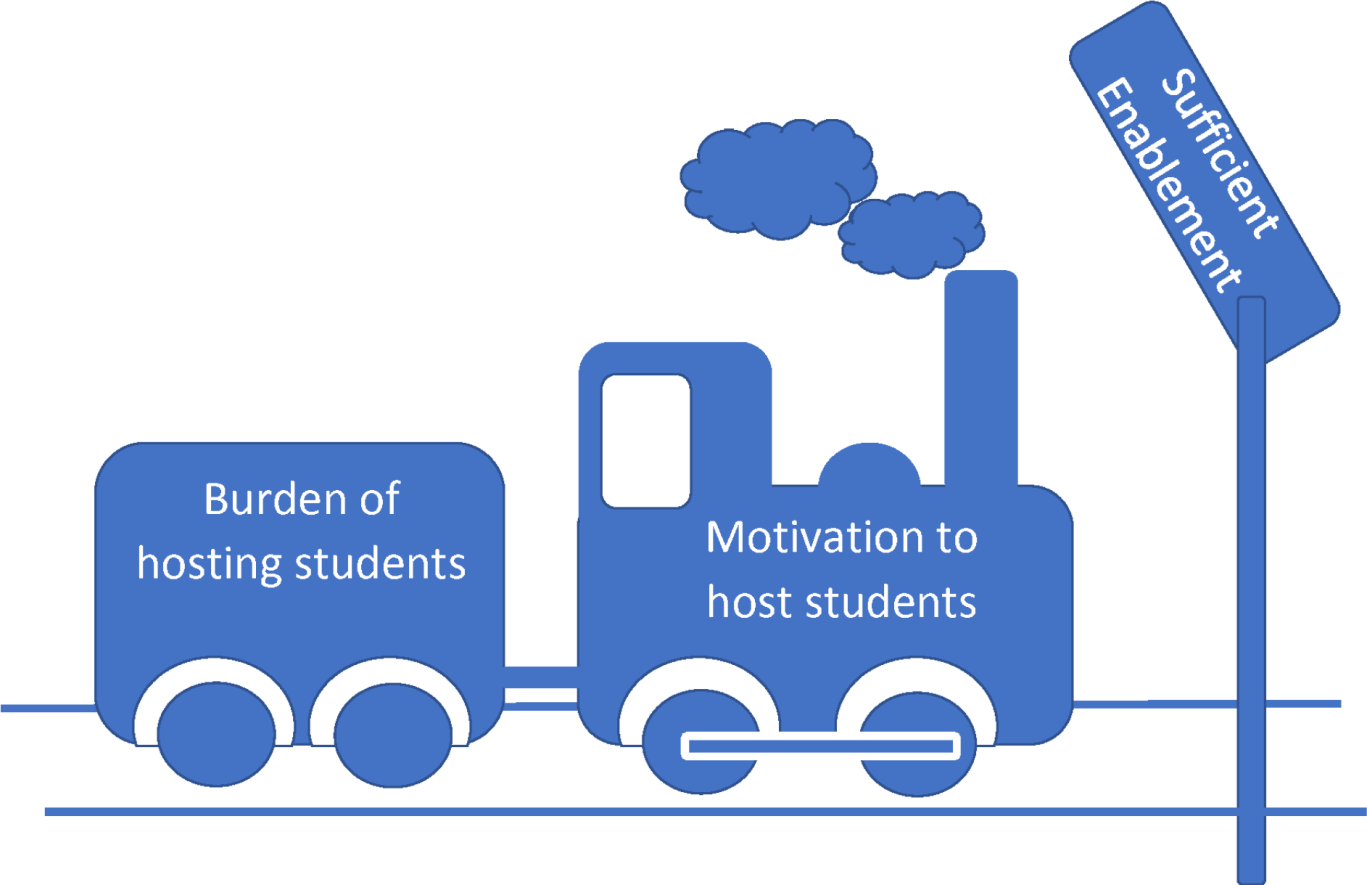
Factors influencing a practice decision to continue hosting medical student placements

**Figure 3.**
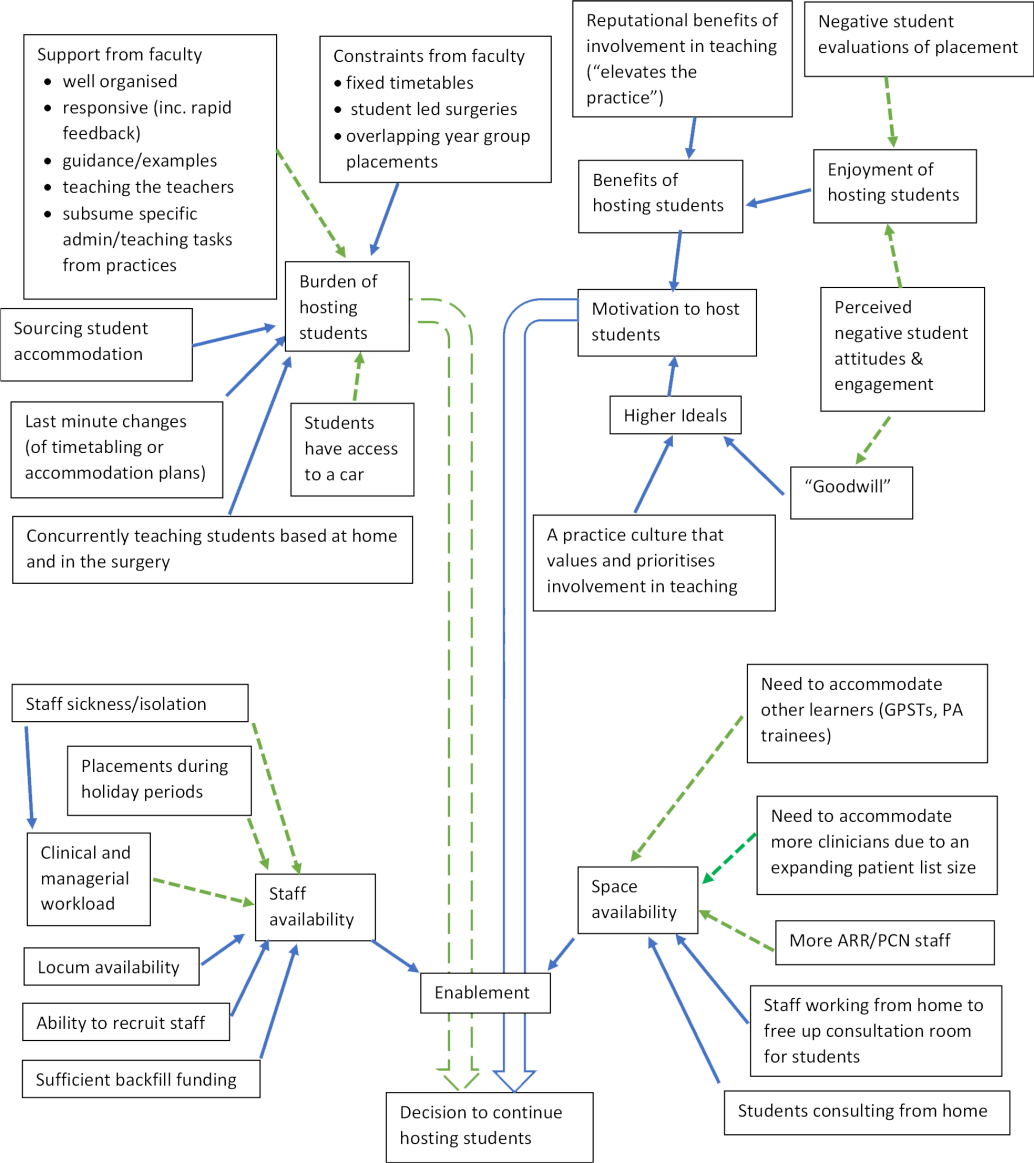
Thematic map of factors influencing a practice continuing to host medical student placements. (Solid blue line indicates an enhancing effect, dashed green line indicates a reductive effect). ARR = additional roles reimbursement. GPSTs = GP specialty trainees. PA = physician associate. PCN = primary care network

These appeared to suggest a model whereby practices host students because motivation overcomes the burden of hosting, but only if a certain level of resource enablement is present (a model represented pictorially in Figure 2). These themes and their constituent sub-themes are described below and they are presented as a thematic map in Figure 3.

#### Enablement

The theme of enablement concerned factors that governed whether or not hosting students was practically possible. It seemed these combined factors produced a stop-and-go outcome as to whether a practice was in a position to be able to host student placements.

The two key elements of enablement appeared to be availability of space and availability of staff.

##### Availability of space

Practices stated that pressure on consulting room space was created by the need to accommodate other learners (specifically trainee GPs and physician associates) and to accommodate greater numbers of clinical staff. Servicing an expanded list size was quoted as one cause of the need to accommodate more clinicians. However, more practices referred to the way in which the need to accommodate new advanced practitioner roles employed by their PCN was exacerbating space pressures:

*'The main issue we have is clinical space — for our clinical staff, the ever-increasing ARRS roles, our two trainees and the students.'* (Practice 088)

One practice stated that in order to free-up consulting rooms for students, they had resorted to some staff working from home. Another responder also suggested that medical students consulting from home might be a way of alleviating this pressure.

##### Staff availability

The importance of adequate financial reimbursement for teaching was commonly referred to in the qualitative data. However, a key finding was that this was generally referred to as an enabling factor rather than a motivating factor (that is, practices don’t teach to make money but money enables them to teach). The phrase 'financial incentive' was used once but its context suggested that the meaning concerned economic viability of backfilling clinicians rather than a financial motivation to teach. Responders described how sufficient reimbursement was necessary to release GPs to teach by backfilling some of their clinical duties. In particular, the degree to which reimbursement was sufficient to fund locums to provide backfill cover was a commonly stated consideration:

*'As a practice we see the importance of educating and training students, however financially and practically does not always make it an easier choice due to employment of locums to cover clinical needs when the regular GPs are supervising students.'* (Practice 39)

However, availability of locums was also mentioned as a factor that influenced ability to host medical student placements, as was being able to recruit enough GPs to a practice.

GP workload was perceived as a threat to teaching owing to both the scale and nature of clinical pressures. In addition, one responder felt that a GP partner’s responsibility is such that they may be more likely than employed staff to have to drop everything and see patients when the practice is in crisis. Staff absence owing to sickness (including post-COVID syndrome), needing to self-isolate, or being on holiday were also described as key challenges to delivering student placements. This was discussed in terms of both its direct effects on having staff available to teach and in terms of its wider effects on clinical and managerial workload. School holiday periods were seen as particularly challenging to placement delivery owing to reduced staffing at those times.

### Motivation to host student placements

The theme of motivation concerned factors that appeared to produce or reduce the drive to continue hosting student placements:

*'We enjoy hosting the students & find it a positive experience, as do our staff & patients.'* (Practice 086)

*'We find ourselves hosting the students increasingly out of goodwill but as we are getting more stressed, and tired, alongside students’ negative attitudes to GP placements, this goodwill is diminishing.'* (Practice 013)

The analysis of responder explanations suggested that practices were motivated by the benefits of hosting students as well as by commitment to a set of higher ideals. On the whole, enjoyment derived from hosting students was the benefit described. This came through strongly from the data, although wider reputational benefits for the practice did get a mention. Higher ideals seemed to concern feelings of goodwill and/or altruism, as well as being part of a practice culture that valued and prioritised delivering high quality teaching.

Negative student attitudes and poor student engagement appeared to be demotivating, owing to the effect on enjoyment derived from hosting students. Indeed, one practice specifically mentioned that this had influenced some of their staff to cease their involvement with teaching students. Negative student attitudes included a perceived undervaluing of primary care, unrealistic expectations of what a practice could deliver, unprofessionalism, inflexibility, and a lack of gratitude or understanding for being hosted despite adverse practice circumstances. Student engagement was described in terms of level of interest, motivation, and punctuality, and was perceived by practices as deteriorating close to exams. Negative student evaluation of a placement also appeared to be demotivating, particularly (as stated by one responder) when it was given by a student who had displayed attitudinal issues. One responder also discussed the impact that negative student attitudes can have on the feeling of goodwill that had been driving teaching at the practice.

### The burden of hosting student placements

Responders commonly mentioned factors that could increase or reduce the degree to which hosting medical students felt burdensome to the practice. Sourcing accommodation was quoted as burdensome, especially when budget limits were unrealistic or in very rural locations where options were limited. Last-minute changes to student plans were also perceived as increasing the burden to practices. Examples included the need to reorganise or re-timetable at short notice owing to students self-isolating, or the need to source accommodation at short notice owing to students changing their travel plans. Meeting the needs of students isolating at home concurrently with those based in the surgery was also perceived as burdensome:

*'Lots of issues with students being off with positive COVID tests and then having to organise remote consulting* [from home] *last minute. Also issues with trying to find accommodation last minute when transport plans change.'* (Practice 008)

Constraints placed by central faculty also appeared to have the potential to be perceived as burdensome. These included faculty stipulations regarding timetabling and learning activities (for example, mandating a certain number of student-led surgeries) as well as faculty establishing overlapping placement dates for different year groups. However, support from central faculty was also perceived to have the potential to reduce the burden of hosting placements. Support from faculty that was appreciated or suggested included the following: being well organised and responsive (including rapid turnaround of student placement evaluations); provision of practical written guidance and examples; educator support or development sessions; and faculty subsuming certain administrative and teaching tasks currently delivered by practices (for example, arranging accommodation, teaching self-isolating students). Students having access to a car was also perceived as making placement provision easier.

## Discussion

### Summary

In the population of practices studied, by far the main stated challenges to continuing hosting medical students are clinical/practice workload and lack of physical space (being picked by 81% and 55% of practices, respectively). Below these, the next most commonly perceived challenges were student absence owing to illness or isolating, lack of clinical staff in the practice, and the need to educate other learners hosted by the practice (picked by 38%, 34%, and 31% of practices, respectively).

Thematic analysis of free-text responses elicited a model whereby practices host students because motivation to teach overcomes the burden of hosting, but only if a certain level of resource enablement is present. The primary motivator for hosting medical students was enjoyment, although reputational benefits and being motivated by higher ideals were also noted. Enabling factors that needed to be sufficiently present for practices to host students were the availability of space and of staff (the latter including clinical/practice workload and backfill funding). Space pressures were perceived as being exacerbated by the need to accommodate more clinicians, especially advanced practitioners employed by PCNs under the ARRS.

### Strengths and limitations

This study is the first to provide a clear picture of the scale and prevalence of the different threats to undergraduate primary care teaching capacity. The 95% response rate means there is little chance of these findings being affected by response bias. The study population of 120 practices is limited to the East of England and defined by hosting students for one specific medical school. However, it is believed this population is sufficiently large, dispersed, and heterogeneous in terms of practice size, patient demographic, and geographical context (urban–rural, deprived–affluent, near–far) to make these findings generalisable. Furthermore, the excellent 95% response rate may not have been achievable if the study population had included other regions or medical schools.

In terms of reflexivity, RD is an academic GP with responsibility for their medical school’s undergraduate GP teaching and SA is a medical student who has just completed their penultimate year of study. This positioning may have influenced their interpretation of the qualitative data and subsequent thematic analysis.

### Comparison with existing literature

Lack of premises space has previously been identified as one of many barriers to medical student teaching in primary care.^1,4^ However, the study provides the first evidence of the scale and prevalence of this threat to undergraduate teaching capacity (being the most commonly picked challenge after workload). Given that Cambridge medical school places students in groups of two (with practices having the option to increase to four) these space issues may be even more acute in regions where medical students are placed in larger groups than this. As such, it adds weight to previous recommendations that capital investment in primary care premises is urgently needed in order to train the workforce of the future.^1^ Medical students consulting from home has been suggested as a possible holding measure while this is awaited.^13^

The findings suggest that some recent changes to primary care appear to have presented threats to medical student teaching more than others. The need to educate or supervise other learners was the fifth most commonly picked challenge (*n* = 35/114 practices), which is a higher frequency than if answers had been selected at random (*n* = 28.5/114). This result may be related in part to the 25% increase in GP trainee numbers over the preceding 3-year period.^9^ Conversely, the shift to remote consulting was only picked by 28 practices, which suggests that this development is less of a threat to placement capacity. Furthermore, the qualitative data strongly indicate that the recent ARRS has exacerbated the lack of space for hosting medical students. This fits with published reports highlighting how funding for PCN-employed ARR staff has not been accompanied by funding of space to accommodate them.^8^

In the study, the level of financial reimbursement for teaching was not one of the options most commonly selected (chosen by 28 of 114 practices). At first sight this result may appear to contradict the considerable body of evidence concerning the relative underfunding of medical student teaching in primary care^1,14^. However, this result is probably not generalisable owing to the considerable regional variation in funding that existed in England before the introduction of national funding arrangements in September 2022.^15^ Although these arrangements were introduced to address underfunding, it is possible that this problem may have been less acute in the region studied compared with other regions. However, with UK inflation predicted to reach 15% by 2023,^16^ underfunding could quickly become an issue again, given the exposure of undergraduate GP teaching to market cost pressures (such as locum fees or overnight student accommodation).^15^

The thematic analysis confirms the findings of other studies that enjoyment was the main motivator for clinician involvement in teaching,^17^ and one which can be reduced by perceived negative student attitudes.^18^ While only 17 of 114 practices in the study judged student attitudinal issues to be one of their top four challenges to delivering placements, the data did contain at least one example of this causing clinicians to withdraw from teaching. Previous studies have uncovered further intrinsic motivators that were not found in the present study (for example, feeling valued by learners and faculty^18^ and identifying as part of a community of educators).^4^ This would suggest the qualitative data did not saturate.

Much of the literature concerning the factors influencing GPs’ decisions whether or not to teach is framed in terms of facilitators and barriers.^4^ However, recent research by Wisener *et al* ^18^ found that such factors cannot be viewed in a binary fashion. The thematic map supports a non-binary view. This is because the present study's analysis finds the decision to continue hosting student placements as being influenced by the interplay of three dimensions: enablement, motivation to host students, and burden of hosting placements. Furthermore, Wisener *et al* found that while certain factors (such as money) were not motivating factors, if they deteriorated to a certain tipping point, the clinician would cease involvement in teaching. Again, this supports the model suggested by the present study's analysis whereby certain enabling factors must reach a threshold in order for motivation to have an effect.

### Implications for research and practice

Relative underfunding of medical student teaching in primary care has recently been addressed in England.^15^ Clinical workload and lack of space now appear to be by far the biggest threats to the future of medical student teaching capacity in primary care. Without urgent action, it is likely that these will deteriorate to the point of breaching a lower threshold of enablement in many GP practices. Such withdrawal of practices from teaching could precipitate a 'death spiral', whereby the level of medical student experience in general practice that is needed to drive their uptake of GP careers^3^ becomes insufficient, thereby reducing further the number of GPs available to teach in primary care. Furthermore, funding to teach needs to keep pace with inflation or this will once again become a major barrier and potentiate the deteriorating levels of enablement to teach that is already being experienced by many practices.

Medical schools should do what they can to minimise the burden of hosting students, to maximise the enjoyment of hosting students, and to address negative student attitudes related to learning medicine in primary care. The present study's qualitative data, as well as the literature on motivation to teach,^4,18^ can inform this work. However, the primary care workload and space crises appear to be by far the greatest threat, and seem to have a threshold effect. If these crises are not addressed, then the point may be reached where any actions taken to safeguard educator motivation, and to minimise the burden of teaching, are unlikely to influence the decision whether to host students.

## Data

The dataset relied on in this article is available from the corresponding author on reasonable request.
